# Nonhealing scrotal ulceration—an unusual manifestation of TB epididymo‐orchitis: case report and review of literature

**DOI:** 10.1002/ccr3.1313

**Published:** 2017-12-05

**Authors:** Umesh Jayarajah, Pamathy Gnanaselvam, Sivasuriya Sivaganesh

**Affiliations:** ^1^ University Surgical Unit National Hospital of Sri Lanka Colombo Sri Lanka; ^2^ Department of Surgery Faculty of Medicine University of Colombo Colombo Sri Lanka

**Keywords:** Case report, chronic epididymo‐orchitis, genitourinary tuberculosis, scrotal ulcers

## Abstract

The clinical presentation of genitourinary tuberculosis (TB) may be variable and a high index of suspicion is required for a timely diagnosis, especially in endemic areas. Recurrent scrotal ulcers associated with epididymo‐orchitis even without other constitutional symptoms should alert the clinician of a possible diagnosis of TB.

## Background

Extrapulmonary tuberculosis (TB) though rare in the Western setting is quite prevalent in South Asia. Genitourinary TB accounts for 15–20% of all extrapulmonary cases of TB 1, 2. The clinical features of genitourinary TB are not always discernible from other pyogenic infections and inflammatory disorders in the first instance. Atypical symptoms, chronicity, and poor response to standard measures indicate the possibility of TB even in the absence of systemic manifestations. This is particularly so in regions where TB is endemic like South Asia and where migrant South Asian and African communities reside in the West. The authors present an unusual case of TB epididymo‐orchitis presenting with nonhealing scrotal ulcers.

## Case Presentation

A 46‐year‐old male farmer, presented with a history of multiple, recurrent discharging scrotal ulcers over a period of three (3) years. He had had multiple wound debridements and dressings done, but the ulcers had recurred a few months after complete healing. The discharging wounds occurred after the formation and subsequent rupture of pustules.

**Figure 1:**
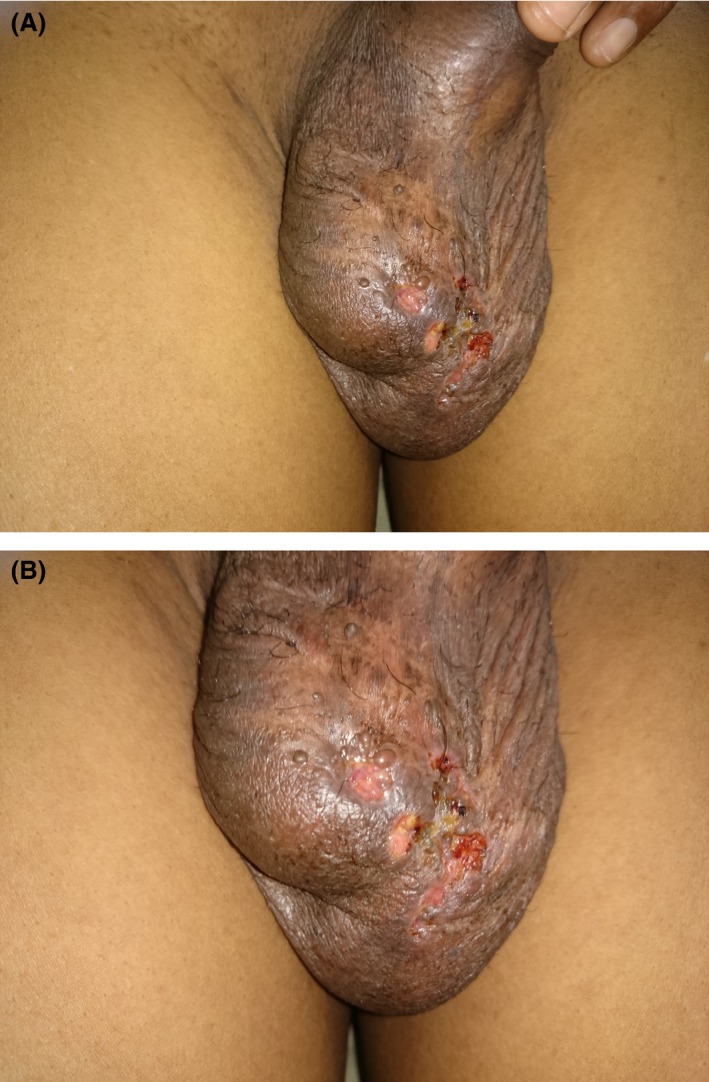
(A and B) The images show multiple, superficial, noninfected scrotal ulcers.

He was otherwise healthy with no constitutional symptoms, significant comorbidities, and no previous history or contact history of TB. He had received the BCG vaccination during infancy. He was an averagely built male with normal general and systemic examination. Genitourinary examination revealed bilateral craggy, hard epididymes, an enlarged and tender right testis, and multiple, superficial, noninfected scrotal ulcers (Fig. 1A and B).

Ultrasonography showed bilateral normal‐sized testes. The right epididymis was enlarged and thickened with a 1.4 × 1 cm sized hypoechoic lesion in relation to the head of the right epididymis and a 2 cm × 1.8 cm sized hypoechoic lesion in relation to the lower pole of the right testis. Internal vascularity was noted. The right testis was not identified separately from the lesion. Furthermore, there was a 1.3 × 0.8 cm sized hypoechoic lesion in the left testis and a 0.7 × 0.6 cm sized hypoechoic lesion in relation to the left epididymis.

His complete blood count and chest radiograph were normal. Erythrocyte sedimentation rate (ESR) was 90 mm/h, and the Mantoux was strongly positive with a diameter of 20 mm after 72 h. The urine full report (UFR) showed a field full of pus cells, and routine bacterial cultures were negative.

**Figure 2:**
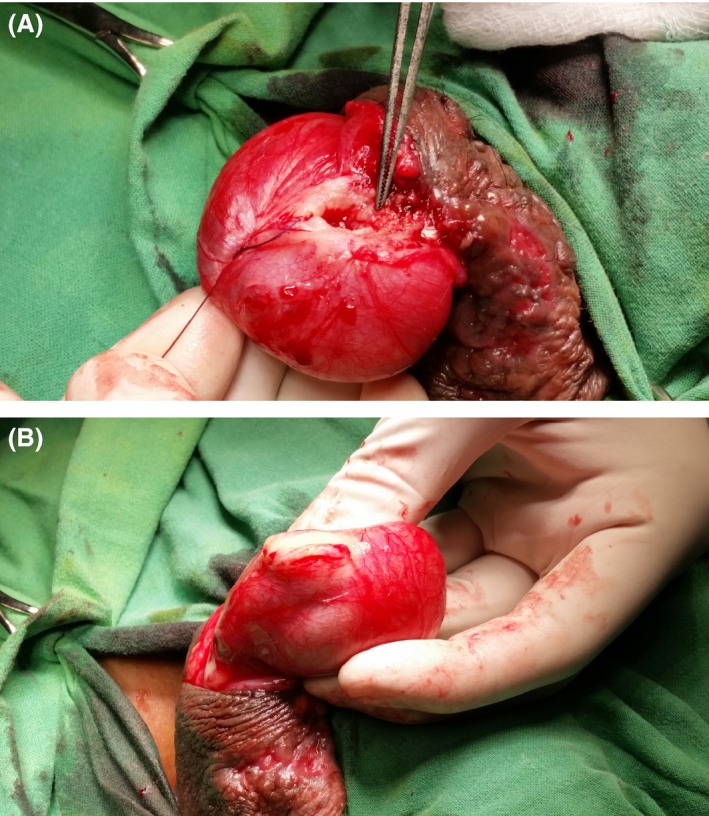
(A and B) Intraoperative findings: enlarged right epididymis which was irregular and thickened with mild enlargement of the right testis with an irregular surface lesion, favoring chronic epididymo‐orchitis.

He underwent surgical exploration where the right epididymis was noted to be enlarged, irregular, and thickened with mild enlargement of the right testis with an irregular surface lesion, favoring chronic epididymo‐orchitis (Fig. 2A and B). A right epididymal and testicular biopsy was performed, and histology revealed evidence of granulomatous inflammation with central caseous necrosis favoring mycobacterial TB infection. There was no evidence of intratubular germ cell neoplasia or malignancy. The Ziehl–Neelsen stain was negative for acid‐fast bacilli. Pus from the scrotal discharge, the biopsy specimen, and the urine sent for TB culture returned negative.

He was started on category 1 antituberculosis therapy, that is, quadruple antimicrobial therapy for 2 months followed by dual therapy with rifampicin and isoniazid for 4 months, for which there was a good response with healing of the scrotal ulcers. He was compliant, and no significant adverse effects of treatment were noted. However, three (3) months into therapy, he presented with worsening lower urinary tract symptoms, strangury, and acute urinary retention. He required suprapubic catheterization, and subsequent retrograde urethrography revealed a bulbar urethral stricture for which he is under urological care.

## Discussion

Tuberculosis (TB) is a major global health problem. TB ranks alongside the human immunodeficiency virus (HIV) as a leading cause of death worldwide, particularly in developing countries 3. In 2014, the prevalence of bacteriologically confirmed TB was 531 (421–655) per 100,000 population; the prevalence of clinically diagnosed TB (i.e., smear‐negative and culture‐negative TB, including all extrapulmonary cases) was 116 (91–143) per 100,000 population; the prevalence of extrapulmonary TB was 58 (43–75) per 100,000 population 3. Genitourinary tuberculosis is the second most common type of extrapulmonary tuberculosis 4 and constitutes about 15–20% of all extrapulmonary tuberculosis 1, 2. Although hematogenous spread from pulmonary TB is thought to be the source of genitourinary TB, about 30–50% of genital TB had no associated pulmonary TB 5. This patient also had no previous history or contact of TB, and his chest X‐ray was normal. A previous case report which reported a similar clinical presentation also did not exhibit evidence of previous pulmonary TB 1.

Although cases of tuberculous chronic epididymo‐orchitis have been reported, the association of scrotal ulcers is a rare phenomenon, and to the author's knowledge, only one previous report has documented a similar clinical picture 1. However, in our patient, there were no constitutional symptoms unlike in the previously reported case. A retrospective case series of 29 cases of scrotal TB reported by Lee et al. 6 showed that most presented as epididymo‐orchitis in isolation or with a scrotal swelling or lump. However, the association of recurrent scrotal ulcers was not reported in that series. Furthermore, this case illustrates the potentially serious morbidity of developing a urethral stricture in a young male during therapy, probably compounded by the late diagnosis.

The ultrasonography in our patient showed the characteristic hypoechoic lesions described in tuberculous epididymo‐orchitis 1, 7.

The Mantoux response was strongly positive in the reported case. The Mantoux positivity is dependent on multiple factors one of which is previous vaccination, and thus, careful interpretation is necessary. While previous past TB infection and vaccination are common causes for a false‐positive Mantoux, a strong reaction greater than 15 mm, 20 mm in the case of our patient is less likely to be due to the previous immunization 8.

In the above patient, the TB cultures were negative. Although a conclusive microbiological diagnosis cannot be made in the absence of a positive culture, his clinical features were typical of TB epididymo‐orchitis. Furthermore, in addition to histological evidence of a caseating granulomatous lesion compatible with tuberculosis, complete resolution of the scrotal ulcers was noted after starting therapy strengthening the case for a diagnosis of TB.

The negative TB culture is most likely the result of antibiotic therapy he received in several instances before he presented to our unit. Ciprofloxacin, which is a second‐line anti‐TB drug, is commonly prescribed for epididymo‐orchitis and urinary tract infections. Thus, irrational prescription of ciprofloxacin may lead to negative consequences such as antimicrobial resistance to TB and negative cultures resulting in diagnostic dilemma. Therefore, particularly in countries where TB is prevalent, antimicrobials that are used as second‐line drugs or alternative regimes should be used with extreme caution, particularly when TB is a differential diagnosis.

Once the diagnosis is established, treatment consists of quadruple antimicrobial therapy for 2 months followed by dual therapy with rifampicin and isoniazid for a further 4–8 months. Usually, Genitourinary TB responds well to the treatment as the drugs rifampicin and isoniazid penetrate well into the cavitary lesions associated with genitourinary TB. The patient was started on quadruple antimicrobial therapy with isoniazid, rifampicin, ethambutol, and pyrazinamide for 2 months followed by dual therapy with rifampicin and isoniazid for 4 months, which is the standard treatment for newly diagnosed TB in Sri Lanka. In Sri Lanka, the above four drugs and streptomycin are used as first‐line drugs for TB. In cases of recurrences, treatment failures and relapses longer duration of therapy with different antimicrobial combinations are recommended. Second‐line drugs such as ofloxacin, ciprofloxacin, kanamycin, amikacin, capreomycin, ethionamide, prothionamide, and cycloserine are used in multidrug‐resistant TB 9.

Surgical exploration is indicated in tuberculous epididymo‐orchitis if the lesion loses its tenderness while maintaining nodularity, indicating a possible malignancy 10.

## Conclusion

The clinical presentation of TB epididymo‐orchitis can be atypical and a high index of suspicion is required for early diagnosis, especially when dealing with populations living in endemic areas and migrant populations from those regions. Diagnosis is straightforward using ultrasonography, microbiology, and biopsy, and therapy requires prolonged poly antimicrobial use. Follow‐up should include observation for complications.

## Consent for Publication

Informed consent was obtained from the patient for publication.

## Authorship

UJ and GP: contributed to planning, writing of the manuscript, and literature review. SS: contributed to overall design, final editing, and final approval. All authors read and approved the final manuscript.

## Conflict of Interest

None declared.
